# IGF2BP2 Polymorphisms Are Associated with Clinical Characteristics and Development of Oral Cancer

**DOI:** 10.3390/ijms21165662

**Published:** 2020-08-07

**Authors:** Chia-Hsuan Chou, Chien-Yuan Chang, Hsueh-Ju Lu, Min-Chien Hsin, Mu-Kuan Chen, Hsien-Cheng Huang, Chia-Ming Yeh, Chiao-Wen Lin, Shun-Fa Yang

**Affiliations:** 1 Institute of Medicine, Chung Shan Medical University, Taichung 402, Taiwan; wishwing1109@hotmail.com (C.-H.C.); Darong14@gmail.com (C.-Y.C.); sinmusha@hotmail.com (M.-C.H.); 53780@cch.org.tw (M.-K.C.) yehcm0525@outlook.com (C.-M.Y.); 2 Department of Medical Research, Chung Shan Medical University Hospital, Taichung 402, Taiwan; 3 Petite Doris Clinic, Taichung 408, Taiwan; 4 Division of Medical Oncology, Department of Internal Medicine, Chung Shan Medical University Hospital, Taichung 402, Taiwan; hsuehju0311@gmail.com; 5 School of Medicine, Chung Shan Medical University, Taichung 402, Taiwan; 6 Department of Otorhinolaryngology-Head and Neck Surgery, Changhua Christian Hospital, Changhua 500, Taiwan; 7 Cancer Research Center, Changhua Christian Hospital, Changhua 500, Taiwan; 8 Department of Emergency Medicine, Kuang Tien General Hospital, Taichung 433, Taiwan; asiantcumed@gmail.com; 9 Institute of Oral Sciences, Chung Shan Medical University, Taichung 402, Taiwan; 10 Department of Dentistry, Chung Shan Medical University Hospital, Taichung 402, Taiwan

**Keywords:** IGF2BP2, polymorphism, oral cancer, lymph node metastasis

## Abstract

Insulin-like growth factor 2 mRNA-binding protein 2 (IGF2BP2) is associated with insulin resistance, lipid metabolism, and tumorigenesis. However, the association between the IGF2BP2 polymorphism and oral cancer risk remains unclear. We recruited 1349 male patients with oral cancer and 1198 cancer-free controls. Three single nucleotide polymorphisms IGF2BP2 rs11705701, rs4402960, and rs1470579 were assessed using real-time polymerase chain reaction. The results indicate that the male patients with oral cancer and with the rs11705701 GA+AA, rs4402960 GT+TT, and rs1470579 AC+CC genotypes had increased risk of advanced clinical stage, larger tumor, and progression of lymph node metastasis compared with those with wild-type IGF2BP2. Moreover, according to The Cancer Genome Atlas dataset, high expression of the IGF2BP2 gene is associated with poor survival in patients with head and neck squamous cell carcinoma. In conclusion, our results suggest that IGF2BP2 polymorphisms are associated with less favorable oral cancer clinical characteristics.

## 1. Introduction

Oral cancer is the sixth most common malignancy worldwide, and oral squamous cell carcinoma (OSCC) is most the prevalent type [1]. In terms of malignancies among men in Taiwan, OSCC has the fourth highest incidence [2]. Despite the advanced surgical treatments for oral cancer, the 5 year survival rate of oral cancer is only 50% [3]. Therefore, the unchanging survival rate in patients with OSCC underscores the need for better prognostic tools, such as the addition of “Depth of invasion” and “Extranodal extension” in the 8th Edition AJCC Cancer Staging Manual [4] and the identification of new biomarkers.

Insulin-like growth factor 2 mRNA-binding protein 2 (IGF2BP2) is a member of the IGF2 mRNA-binding protein family and is encoded by the *IGF2BP2* gene, which is located on chromosome 3q27 [5,6,7]. Studies have suggested that IGF2BP2 is associated with insulin resistance, lipid metabolism, and tumorigenesis [8,9]. In addition, some studies have reported that the single nucleotide polymorphism (SNP) of *IGF2BP2* is associated with diabetes and non-small-cell lung cancer (NSCLC). Rao et al. [10] and Wu et al. [11] demonstrated that the *IGF2BP2* polymorphism is associated with type 2 diabetes (T2D) and obesity. Chistiakov et al. demonstrated that the *IGF2BP2* rs11705701 A allele is associated with higher T2D risk [12]. Chen et al. suggested that the *IGF2BP2* rs4402960 polymorphism T allele may be a protective factor for decreased susceptibility to NSCLC among women [13]. Tang et al. found that the *IGF2BP2* rs1470579 variant is a protective factor against esophagogastric junction adenocarcinoma [14].

However, the association between the *IGF2BP2* polymorphism and oral cancer risk remains unclear. In this study, we investigated the potential association between genetic variations in *IGF2BP2* SNPs and the development of OSCC. *IGF2BP2* rs11705701, rs4402960, and rs1470579 were selected, and a case–control study was conducted to analyze the relationship between the *IGF2BP2* gene polymorphism and OSCC in a group of Taiwanese patients.

## 2. Results

### 2.1. Characteristics of Study Participants and IGF2BP2 Polymorphism in Oral Cancer

In total, 1349 patients with oral cancer and 1198 healthy controls were included in the final analysis. The patients with oral cancer had significantly higher consumption of betel quid, cigarettes, and alcohol than the healthy controls (Table 1). However, differences in age distribution between the two groups were not statistically significant (*p* = 0.935). To investigate the association between the allele frequency of IGF2BP2 SNPs and the risk of oral cancer, the allele frequencies of IGF2BP2 rs11705701, rs4402960, and rs1470579 in the case and control groups were tested (Table 2). After adjustment for several variables, no significant difference in distributions of IGF2BP2 rs11705701, rs4402960, and rs1470579 was observed between the case and control groups (Table 2).

### 2.2. Association between IGF2BP2 Polymorphic Genotypes and Clinical Features of Oral Cancer

Additionally, we examined the association between the clinical parameters of patients with oral cancer and the IGF2BP2 rs11705701, rs4402960, and rs1470579 polymorphisms. The male patients with oral cancer and the rs11705701 GA+AA, rs4402960 GT+TT, and rs1470579 AC+CC genotypes exhibited higher risk of advanced clinical stage, larger tumor, and progression of lymph node metastasis compared with those with the IGF2BP2 rs11705701 GG, rs4402960 GG, and rs1470579 AA genotypes (Table 3, Table 4 and Table 5).

### 2.3. Association between IGFBP2 mRNA Expression and Clinical Characteristics of Head and Neck Squamous Cell Carcinoma (HNSCC) Tissues from The Cancer Genome Atlas (TCGA) Database

**Figure 1:**
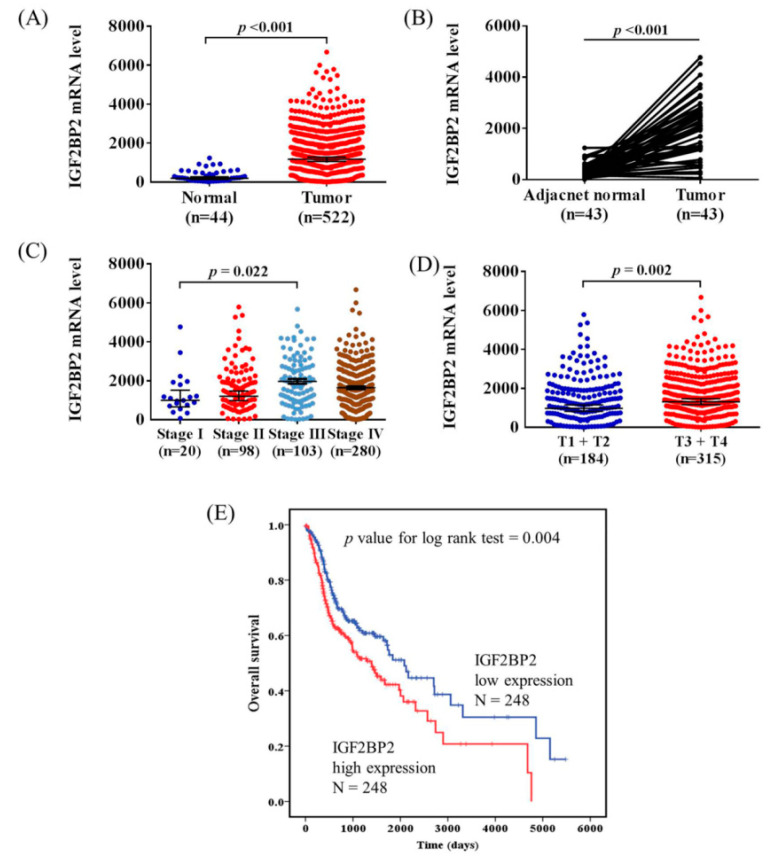
High IGF2BP2 expression was associated with poor survival in patients with head and neck squamous cell carcinoma (HNSCC). (**A**) IGF2BP2 expression in healthy individuals and patients with HNSCC from The Cancer Genome Atlas (TCGA) data portal. (**B**) IGF2BP2 expression in 43 matched tumor tissues and their corresponding noncancerous tissues. (**C**) Comparison of the clinical stage of IGF2BP2 expression. (**D**) Comparison of the tumor T status of IGF2BP2 expression. (**E**) Overall survival analysis of patients with HNSCC with high (red lines) and low (blue lines) IGF2BP2 expression levels. *p* values were determined using a log-rank test.

To further support our findings, we analyzed the cases of head and neck squamous cell carcinoma (HNSCC) from The Cancer Genome Atlas (TCGA) dataset. We found that IGF2BP2 expression was significantly higher in tumor tissues (*p* < 0.001, Figure 1A). In addition, 43 matched tumor tissues and their corresponding noncancerous tissues indicated higher IGF2BP2 expression in the tumors (*p* < 0.001, Figure 1B). Furthermore, the patients with clinical stage III HNSCC had higher IGF2BP2 expression than those in stage I (*p* = 0.022, Figure 1C). IGF2BP2 expression was significantly higher in T3+T4 than in T1+T2 (*p* = 0.002, Figure 1D). Next, we examined the prognosis of HNSCC. A Kaplan–Meier plot indicated that the group with high IGF2BP2 expression had poorer overall survival than the low IGF2BP2 expression group (*p* = 0.004, Figure 1E). These results suggest that IGF2BP2 mRNA expression is related to tumor mass and associated with poor survival in HNSCC patients.

## 3. Discussion

In this study, we found no significant correlations between the development of oral cancer and the *IGF2BP2* rs11705701 G > A, rs4402960 G > T, and rs1470579 A > C SNPs. However, in logistical analyses, we determined that patients with oral cancer and the *IGF2BP2* rs11705701 GA+AA, rs4402960 GT+TT, and rs1470579 AC+CC genotypes had higher risk in terms of clinical stage, tumor size, and lymph node metastasis compared with those with the *IGF2BP2* rs11705701 GG, rs4402960 GG, and rs1470579 AA genotypes.

Studies have reported that IGF2BP2 is amplified and overexpressed in numerous cancers [15,16] and is often associated with poor prognosis [17,18,19,20]. Dai et al. demonstrated that IGF2BP2 promotes malignancy proliferation through its client mRNA IGF2 and high mobility group A1 [21]. Barghash et al. found that IGF2BP2 expression was associated with tumor size, clinical stage, metastasis, and short survival in esophageal adenocarcinoma [22]. Chistiakov et al. suggested that the *IGF2BP2* rs11705701 A allele was associated with significantly increased T2D risk [12]. In our study, among the 1349 patients with oral cancer, having the rs11705701 GA+AA genotype was associated with advanced clinical stage, development of a large tumor, and higher risk of lymph node metastasis. The rs11705701 G > A SNP is located in the 5′ promoter region of the *IGF2BP2* gene and may thus affect promoter activity. Moreover, Chistiakov et al. reported that the *IGF2BP2* G allele may destroy the transcription factors NFIC and ETS1 for binding [12]. This could explain the activating effect of the A allele in IGF2BP2 transcription.

Several case–control studies have reported that IGF2BP2 rs4402960 is associated with cancer progression. Liu et al. revealed that those with the rs4402960 GT+TT genotype or T allele gene had significantly increased susceptibility to breast cancer [23]. Zhang et al. and Huang et al. found that those with the IGF2BP2 rs4402960 T allele had higher risk of T2D, and this polymorphism may also influence therapeutic effects in those of Chinese ethnicity [24,25]. However, due to the lack of information about diabetes mellitus status in our oral cancer patient group, the relationships between IGF2BP2 SNPs and diabetes mellitus in oral cancer patients should be further addressed in the future.

Our data also indicated that advanced clinical stage, development of large tumor, and progression of lymph node metastasis were associated with the *IGF2BP2* rs1470579 AC+CC genotype. These results are consistent with the observation of Huang et al.—that the C allele of *IGF2BP2* rs1470579 was associated with higher risk in Chinese people with T2D [24]. Some studies have reported that the intron region is involved in regulating tissue-specific mRNA transcription and translation [26,27,28,29]. The *IGF2BP2* rs4402960 and rs1470579 polymorphisms are both located in the intron region, which might influence the risk of oral cancer through IGF2BP2 gene post-transcription mechanisms. More research is required to elucidate the functions of the *IGF2BP2* rs4402960 G > T and rs1470579 A > C polymorphisms.

In the present study, an analysis of patients with HNSCC in the TCGA database revealed higher expression of IGF2BP2 in tumors. Overexpression of IGF2BP2 has been reported in several types of solid cancer, such as breast cancer and esophageal adenocarcinoma [17,22]. Furthermore, higher expression of IGF2BP2 was significantly associated with poor prognosis in acute myelocytic leukemia [30]. These data are consistent with our findings using data from the TCGA database; we found that higher IGF2BP2 expression was significantly correlated with poorer survival in HNSCC.

## 4. Materials and Methods

### 4.1. Study Subjects

We recruited 1349 male patients who had been diagnosed with oral cancer at Chung Shan Medical University Hospital in Taichung and Changhua Christian Hospital in Changhua between 2007 and 2019 as the case group. We also selected 1198 cancer-free male controls without a history of cancer from Taiwan Biobank. The study was approved by the Chung Shan Medical University Hospital institutional review board (CSMUH No: CS18235). The medical information of the patients—including tumor–node–metastasis (TNM) clinical staging, primary tumor size, lymph node involvement, and histologic grade—was obtained from their medical records. The patients with oral cancer were clinically staged at the time of their diagnosis according to the TNM staging system of the American Joint Committee on Cancer Staging Manual seventh edition [31]. At the start of the study, whole blood specimens collected from all participants were placed in sterile tubes containing ethylene diamine tetraacetic acid (EDTA), immediately centrifuged, and then stored at −80 °C for later analysis.

### 4.2. DNA Extraction and Genotyping

Genomic DNA was extracted from peripheral blood leukocytes by using QIAamp DNA blood mini kits (Qiagen, Valencia, CA, USA) according to the manufacturer’s instructions. Genomic DNA was dissolved in TE buffer (10 mM Tris and 1 mM EDTA; pH 7.8) and then quantified by measuring the optical density at 260 nm. The final preparation was stored at −20 °C and was used as a template for polymerase chain reaction (PCR). Assessment of allelic discrimination for IGF2BP2 SNPs was performed using a TaqMan assay with an ABI StepOne Real-Time PCR System (Applied Biosystems, Foster City, CA, USA). The results were further analyzed using SDS version 3.0. The total volume of the TaqMan assay mixture was 10 μL, including 5 μL of Genotyping Master Mix, 0.25 μL of probe, and 10 ng of genomic DNA. The real-time PCR reaction involved an initial denaturation step at 95 °C for 10 min, followed by 40 amplification cycles at 95 °C for 15 s and 60 °C for 1 min.

### 4.3. Statistical Analysis

The Mann–Whitney U test and Fisher’s exact test were used to compare demographical characteristics between the oral cancer group and healthy controls. The adjusted odds ratios (AORs)—with 95% CIs of the association between genotype frequency and oral cancer risk as well as clinical pathological characteristics—were measured using multiple logistic regression models after controlling for covariates. A *p* value < 0.05 was considered statistically significant. The data were analyzed using SAS version 9.4 (SAS Institute Inc., Cary, NC, USA).

## 5. Conclusions

In summary, our study investigated the occurrence of the IGF2BP2 polymorphism in Taiwanese patients with oral cancer. Although the rs11705701 G > A, rs4402960 G > T, and rs1470579 A > C polymorphisms of IGF2BP2 did not affect oral cancer susceptibility, our study of Taiwanese men with oral cancer revealed that people with the IGF2BP2 rs11705701 GA+AA, rs4402960 GT+TT, and rs1470579 AC+CC genotypes are more likely to have a more advanced clinical stage, larger tumor, and progression of lymph node metastasis.

## Figures and Tables

**Table 1:** Distributions of demographical characteristics in 1198 controls and 1349 male patients with oral cancer.

Variable	Controls (*n* = 1198)	Patients (*n* = 1349)	*p* Value
Age (yrs)			
≤55	608 (50.8%)	683 (50.6%)	*p* = 0.935
>55	590 (49.2%)	666 (49.4%)	
Betel quid chewing			
No	1000 (83.5%)	342 (25.4%)	
Yes	198 (16.5%)	1007 (74.6%)	*p* < 0.001 *
Cigarette smoking			
No	563 (47.0%)	210 (15.6%)	
Yes	635 (53.0%)	1139 (84.4%)	*p* < 0.001 *
Alcohol drinking			
No	961 (80.2%)	708 (52.5%)	
Yes	237 (19.8%)	641 (47.5%)	*p* < 0.001 *
Stage			
I+II		632 (46.9%)	
III+IV		717 (53.1%)	
Tumor T status			
T1+T2		679 (50.3%)	
T3+T4		670 (49.7%)	
Lymph node status			
N0		890 (66.0%)	
N1+N2+N3		459 (34.0%)	
Metastasis			
M0		1339 (99.3%)	
M1		10 (0.7%)	
Cell differentiation			
Well differentiated		189 (14.0%)	
Moderately or poorly differentiated		1160 (86.0%)	

Mann–Whitney U test or Fisher’s exact test was used to identify differences between healthy controls and patients with oral cancer. * *p* < 0.05.

**Table 2:** Odds ratios and 95% CIs of oral cancer associated with *IGF2BP2* genotypic frequencies.

Variable	Controls (*n* = 1198) (%)	Patients (*n* = 1349) (%)	OR (95% CI)	AOR (95% CI) ^a^
rs11705701				
GG	743 (62.0%)	831 (61.6%)	1.000 (reference)	1.000 (reference)
GA	390 (32.5%)	466 (34.5%)	1.068 (0.904–1.262)	1.063 (0.864–1.308)
AA	65 (5.5%)	52 (3.9%)	0.715 (0.490–1.044)	0.778 (0.486–1.247)
GA+AA	455 (38.0%)	518 (38.4%)	1.016 (0.865–1.192)	1.024 (0.839–1.249)
rs4402960				
GG	710 (59.3%)	799 (59.2%)	1.000 (reference)	1.000 (reference)
GT	413 (34.5%)	483 (35.8%)	1.039 (0.880–1.227)	1.123 (0.914–1.380)
TT	75 (6.2%)	67 (5.0%)	0.793 (0.562–1.120)	0.777 (0.505–1.194)
GT+TT	488 (40.7%)	550 (40.8%)	0.999 (0.853–1.171)	1.068 (0.877–1.300)
rs1470579				
AA	689 (57.5%)	779 (57.7%)	1.000 (reference)	1.000 (reference)
AC	428 (35.7%)	499 (37.0%)	1.031 (0.874–1.216)	1.121 (0.913–1.376)
CC	81 (6.8%)	71 (5.3%)	0.775 (0.554–1.083)	0.768 (0.505–1.166)
AC+CC	509 (42.5%)	570 (42.3%)	0.989 (0.845–1.157)	1.063 (0.874–1.293)

The odds ratio (ORs) with their 95% CIs was estimated using logistic regression models. ^a^ The adjusted odds ratios (AORs) with their 95% CIs were estimated using multiple logistic regression models after controlling for age and consumption of betel quid, cigarettes, and alcohol.

**Table 3:** Odds ratios and 95% CIs of clinical status associated with genotypic frequencies of *IGF2BP2* rs11705701 in male patients with oral cancer (*n* = 1349).

Variable			AOR (95% CI)	*p* Value
Clinical Stage				
rs11705701	Stage I+II	Stage III+IV		
GG	411 (65.0%)	420 (58.6%)	1.00	
GA+AA	221 (35.0%)	297 (41.4%)	1.322 (1.059–1.650)	*p* = 0.014 *
Tumor size				
rs11705701	≤T2	>T2		
GG	436 (64.2%)	395 (59.0%)	1.00	
GA+AA	243 (35.8%)	275 (41.0%)	1.253 (1.004–1.562)	*p* = 0.046 *
Lymph node metastasis				
rs11705701	No	Yes		
GG	574 (64.5%)	257 (56.0%)	1.00	
GA+AA	316 (35.5%)	202 (44.0%)	1.440 (1.143–1.815)	*p* = 0.002 *
Metastasis				
rs11705701	M0	M1		
GG	825 (61.6%)	6 (60.0%)	1.00	
GA+AA	514 (38.4%)	4 (40.0%)	1.078 (0.301–3.854)	*p* = 0.909
Cell differentiated grade				
rs11705701	≤Grade I	>Grade I		
GG	120 (63.5%)	711 (61.3%)	1.00	
GA+AA	69 (36.5%)	449 (38.7%)	1.099 (0.798–1.514)	*p* = 0.562

The adjusted odds ratios (AORs) with their 95% CIs were estimated using multiple logistic regression models after controlling for age and consumption of betel quid, cigarettes, and alcohol. * *p* < 0.05.

**Table 4:** Odds ratios and 95% CIs of clinical status associated with genotypic frequencies of *IGF2BP2* rs4402960 in male patients with oral cancer (*n* = 1349).

Variable			AOR (95% CI)	*p* Value
Clinical Stage				
rs4402960	Stage I+II	Stage III+IV		
GG	392 (62.0%)	407 (56.8%)	1.00	
GT+TT	240 (38.0%)	310 (43.2%)	1.256 (1.009–1.563)	*p* = 0.042 *
Tumor size				
rs4402960	≤T2	>T2		
GG	427 (62.9%)	372 (55.5%)	1.00	
GT+TT	252 (37.1%)	298 (44.5%)	1.350 (1.085–1.680)	*p* = 0.007 *
Lymph node metastasis				
rs4402960	No	Yes		
GG	549 (61.7%)	250 (54.5%)	1.00	
GT+TT	341 (38.3%)	209 (45.5%)	1.360 (1.081–1.711)	*p* = 0.009 *
Metastasis				
rs4402960	M0	M1		
GG	794 (59.3%)	5 (50.0%)	1.00	
GT+TT	545 (40.7%)	5 (50.0%)	1.439 (0.413–5.013)	*p* = 0.568
Cell differentiated grade				
rs4402960	≤Grade I	>Grade I		
GG	121 (64.0%)	678 (58.5%)	1.00	
GT+TT	68 (36.0%)	482 (41.5%)	1.260 (0.914–1.735)	*p* = 0.158

The adjusted odds ratios (AORs) with their 95% CIs were estimated using multiple logistic regression models after controlling for age and consumption of betel quid, cigarettes, and alcohol. * *p* < 0.05.

**Table 5:** Odds ratios and 95% CIs of clinical status associated with genotypic frequencies of *IGF2BP2* rs1470579 in male patients with oral cancer (*n* = 1349).

Variable			AOR (95% CI)	*p* Value
Clinical Stage				
rs1470579	Stage I+II	Stage III+IV		
AA	386 (61.1%)	393 (54.8%)	1.00	
AC+CC	246 (38.9%)	324 (45.2%)	1.306 (1.050–1.624)	*p* = 0.017 *
Tumor size				
rs1470579	≤T2	>T2		
AA	415 (61.1%)	364 (54.3%)	1.00	
AC+CC	264 (38.9%)	306 (45.7%)	1.317 (1.060–1.637)	*p* = 0.013 *
Lymph node metastasis				
rs1470579	No	Yes		
AA	539 (60.6%)	240 (52.3%)	1.00	
AC+CC	351 (39.4%)	219 (47.7%)	1.416 (1.127–1.780)	*p* = 0.003 *
Metastasis				
rs1470579	M0	M1		
AA	774 (57.8%)	5 (50.0%)	1.00	
AC+CC	565 (42.2%)	5 (50.0%)	1.348 (0.387–4.694)	*p* = 0.639
Cell differentiated grade				
rs1470579	≤ Grade I	>Grade I		
AA	115 (60.9%)	664 (57.2%)	1.00	
AC+CC	74 (39.1%)	496 (42.8%)	1.158 (0.844–1.587)	*p* = 0.363

The adjusted odds ratios (AORs) with their 95% CIs were estimated using multiple logistic regression models after controlling for age and consumption of betel quid, cigarettes, and alcohol. * *p* < 0.05.
